# Population structure, dispersion patterns and genetic diversity of two major invasive and commensal zoonotic disease hosts (*Rattus norvegicus* and *Rattus tanezumi*) from the southeastern coast of China

**DOI:** 10.3389/fgene.2023.1174584

**Published:** 2024-01-08

**Authors:** Jiaqiao Li, Enjiong Huang, Yifan Wu, Changqiang Zhu, Wenhao Li, Lele Ai, Qinghua Xie, Zhi Tian, Weiwen Zhong, Gang Sun, Lingling Zhang, Weilong Tan

**Affiliations:** ^1^ Nanjing Bioengineering (Gene) Technology Center for Medicines, Nanjing, China; ^2^ School of Resources and Chemical Engineering, Sanming University, Sanming, China; ^3^ Fujian Agriculture and Forestry University, Fuzhou, China; ^4^ Technology Center of Fuzhou Customs, Fuzhou, Fujian, China; ^5^ Center for Disease Control and Prevention, Longquan, Zhejiang, China

**Keywords:** *R. norvegicus*, *R. tanezumi*, population Genetics, haplotype, microsatellite loci, southeast coast of China

## Abstract

**Background:** The invasive brownrat (*Rattus norvegicus*) and the Oriental rats (*Rattus tanezumi*) are common commensal murid that are important hosts for rodent-borne diseases in southeast Asia. Understanding their population structure and genetic diversity is essential to uncover their invasion biology and distribution dynamics that are essential for controlling rodent-borne diseases.

**Methods:** TA total of 103 *R. norvegicus* and 85 *R. tanezumi* were collected from 13 to 9 coastal areas of six provincial monitoring sentinel sites, respectivelyto assess patterns in their microsatellite loci and their mitochondrial coxl gene region.

**Results:** Eleven sampled populations of *R. norvegicus* were divided into two major clusters by region. The observed heterozygosity values of all regional populations were smaller than expected genetic diversity heterozygosity values and deviated from Hardy-Weinberg equilibrium Nine sample populations of *R. tanezumi* were divided into three clusters; two that included sample from Hainan and Fujian provinces, and one that included samples from the other provinces and cities. The genetic diversity of *R. tanezumi* was highest in samples from Jiangsu and Guangdong provinces.

**Conclusion:** The data in this paper confirm the two invasive rodent species from the southeastern coastal region of China may have relied on maritime transport to spread from the southern region of China to the Yangtze River basin. *R. tanezumi* may then hanve migrated unidirectionally, along the southeastern provinces of China towards the north, while *R. norvegicus* spread in a complex and multidirectional manner in Hainan, Fujian, Zhejiang and Jiangsu Provinces of the country.

## Introduction

Rats are the most adaptable and widespread mammals, that live close to humans and are associated with human migrations (Matisoo-Smith and Robins, 2004; Suzuki et al., 2013). Generally, four major murid rodent species are invasive and commensal, namely, *Rattus rattus*, *Rattus tanezumi*, *Rattus exulans*, and *Rattus norvegicus* (Puckett et al., 2020). *Rattus tanezumi* and *R. norvegicus* serve as hosts for numerous pathogens, representing a significant threat to human health due to their presence. (Himsworth et al., 2013). They are also major pests in farmlands in southern and southwestern China, causing damage to crops during spring and autumn before harvest (Htwe et al., 2012).

In China, the distribution range of *R*. *tanezumi* and *R. norvegicus* has expanded extensively over the past decades (Wang, 2003; Mora et al., 2010). In the 1950s, *R. tanezumi* in China was mainly distributed in the Yangtze River basin, eastern and southern regions (Robins et al., 2008; Deinum et al., 2015). Due to the rapid changes in transportation and the landscape, the geographic distribution of *R. tanezumi* has increased significantly in recent years (Guo et al., 2019). So far, *R. tanezumi* has been reported in Qinghai, Tibet Autonomous Region, west of Xinjiang Uyghur Autonomous Region, north of Hebei and Liaoning (Hou and Jiang, 2008; Ma et al., 2011; Yang et al., 2011). *Rattus norvegicus* is widely distributed in all provinces except Tibet due to its excellent long-distance transmission ability (Wang et al., 2021).

The origin and genetic evolution of the *R. norvegicus* has been controversial. The regions of Eastern Central Asia, including present-day China and Mongolia, have long been regarded as its birthplace. Concurrently, an alternative hypothesis proposes that *R. norvegicus* originated in the realms of southeastern Siberia and northeastern China, as suggested by Lin et al. (2012) (Lin et al., 2012), subsequently embarking on a westward journey across the vast Eurasian grasslands and ultimately reaching Europe. (Gibbs et al., 2004). Recent advancements in fossil dating have unveiled a more illuminating perspective. These cutting-edge analyses contribute to a more robust narrative, indicating that the species originated in southwestern China approximately 1.2–1.6 million years ago. (Jin et al., 2008; Wu and Wang, 2012).

Emerging and re-emerging infectious diseases endanger human health and public safety, and their infections are influenced by the rate by transmission of their natural hosts between geographic areas (Morens et al., 2004). *Rats* as typical zoonotic hosts, harbour ectoparasites such as ticks, lice and mites, and a variety of internal parasites (Puckett et al., 2020). In addition, they harbourrange of bacteria, rickettsiae and pathogens (Huang et al., 2013; Yin et al., 2021). Currently, they are known to be vectors of pathogens of plague (caused by *Yersinia pestis*), leptospirosis (*Leptospira*), hemorrhagic fever with renal syndrome (Hantavirus), COVID-19 (SARS-CoV-2), and scrub typhus (Plyusnina et al., 2009; Blasdell et al., 2011). Among these, plague has had three major outbreaks in human history. Europe was devastated by Justinian’s plague (541–767 AD) and the Black Death (1346–18th Century) (Pollitzer, 1951). In China, tens of people of individuals died of plague in the 19th Century (Morelli et al., 2010). Plague is carried by rodents and spread by the bite of infected fleas, and owing to the endeavors of rodents, notably those thriving in close proximity to human habitats, such as *R. tanezumi* and *R. norvegicus*, ubiquitous in the human environment and frequently interacting with humans in their daily routines, there is a significant escalation in the potential for ticks harbored on these rodents to transmit diseases within human populations. Rodent population dynamics and distribution may also affect the risk of human hantavirus infections in both (Xiao et al., 2018) developing or developed countries (Pimentel et al., 2000). To prevent and monitor the risk posed by the migration of these invasive and commensal rodents, their population genetic structure can be assessed by commonly used molecular genetic markerssuch as among which microsatellite loci and mtDNA as indicators of genetic diversity and phylogenetic relationships (Brown et al., 1979; Montagutelli, 1991). For instance, Zhao et al. (2014) illuminated, via mitochondrial DNA sequencing of the brown house mouse (*R. norvegicus*), that rivers had negligible impact on the genetic patterns of the species (Zhao et al., 2016). In a similar vein, Guo et al. (2019) demonstrated, utilizing microsatellite markers and mitochondrial DNA (COI and D-loop) sequence analyses, that populations of the yellow marmot from Tibet and Sichuan exhibited a linkage transmitted via the Sichuan-Tibet Motorway, as opposed to being introduced via the Tea Horse Road (Guo et al., 2019).

To investigate dispersal patterns and population structure of *R*. *tanezumi and R. norvegicus* along the southeastern coastal areas of China, their populations from six provinces were sampled and screened using microsatellite loci and mtDNA sequences, respectively. The microsatellite loci and mtDNA employed in this study have been previously documented for their informative genetic polymorphism in various studies. These markers have been utilized to analyze the genetic structure and information of mouse populations in regions such as the United States and Asia. (Kunieda et al., 1992; Lack et al., 2012; Guo et al., 2019). Based on the genetic diversity and population structure among each populations, the dispersal patterns of populations were evaluated.

As challenges like mounting industrialization and global warming persist, a growing impact is observed on diverse commensal rodent species. Consequently, we posit that the genetic structure and migration patterns of these two commensal rodents in the coastal regions of China are probably influenced by human activities or transportation mechanisms, reflecting the dynamics within the framework of contemporary society. Our results will provide insights into genetic diversity, genetic differentiation, gene flow patterns, and possible hybridization among populations of different regions, with implications on the potential control of these problem invasive and commensal murid rodents.

## Materials and methods

### Sample collection

**FIGURE 1:**
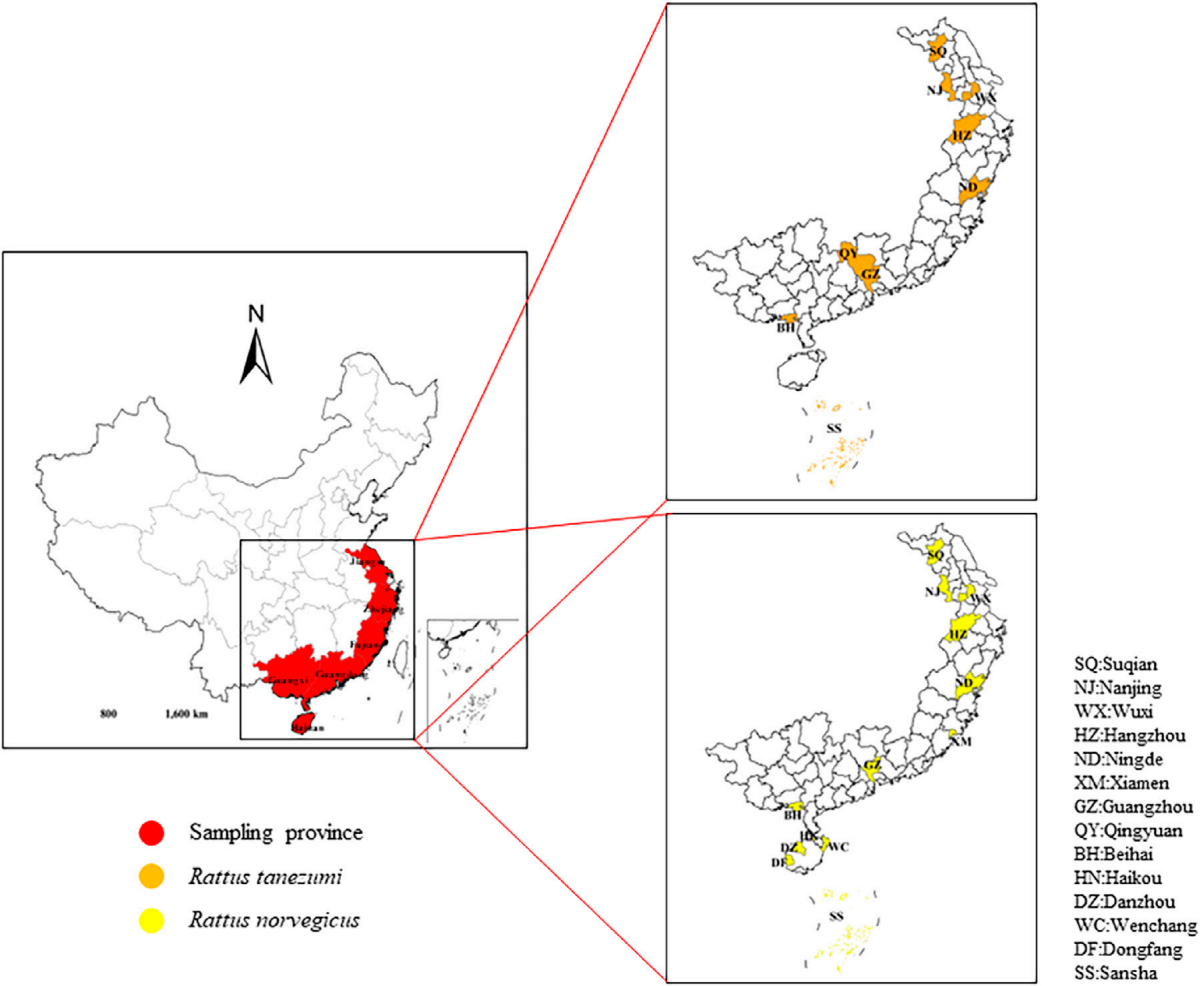
Sampling loca lities. Sampling sites of *Rattus norvegicus* and R. tanezumi from different regions southeastern coast of China. The sampling areas are marked with different colors.

In this study, *R. norvegicus* were sample from 13 localities and *R. tanezumi* from 9 localities in six provinces along the southeastern coast of China (Table 1; Figure 1). Given that it was necessary to avoid factors of kinship among samples, we randomly plac trapping cages and sticky boards in residential areas, ports and other areas at a distance of more than 2 km between sample sites (All captured rodent samples were executed by spinal subluxation (Ethics Committee Batch No: 2018001003)). Muscle and liver tissues were dissected out and frozen at −80 °C. DNA was extracted from mouse samples using the TaKaRa MiniBEST Universal Genomic DNA kit according to the manufacturer’s requirements. OD260/OD280 values and nucleic acid concentrations were recorded in a spectrophotometer (KAIAO,K5600,Beijing, China**)**to determine the concentration and purity of the DNA. The species were genetically-identified based on the screening of the cytochrome (*cytb*)gene region (S1 Table), leading to 88 samples being identified as *Rtanezumi* and 103 as *R. norvegicus*.

**TABLE 1:** Sampling localities and their geographic coordinates and habitat types of rat samples from the southeastern coast of China.

Location	Sample		Code	Geographic coordinate	Habitat type
	*Rattus norvegicus*	*Rattus tanezumi*			
Jiangsu, Suqian	2	15	JS,SQ	33° 47' 2.287" N, 118° 31' 12.900" E	Rural
Jiangsu, Nanjin	1	6	JS,NJ	31° 55' 40.060" N, 118° 50' 34.053" E	Harbor
Jiangsu, Wuxi	14	15	JS,WX	31° 31' 37.947" N, 120° 4' 35.570" E	Harbor
Zhejiang, Hangzhou	15	2	ZJ,HZ	29° 53' 59.378" N, 119° 28' 15.382" E	Urban
Fujian, Nningde	11	7	FJ,ND	26° 58' 41.313" N, 119° 27' 50.081" E	Urban
Fujian, Xiamen	9	–	FJ,XM	24° 41' 51.569" N, 118° 6' 42.614" E	Urban
Guangdong, Guangzhou	15	6	GD,GZ	23° 21' 15.431" N, 113° 32' 5.546" E	Urban
Guangdong, qingyuan	-	15	GD,QY	111° 59' 41.463" N, 111° 59' 41.463" E	Urban
Guangxi, Beihai	15	15	GX,BH	109° 20' 9.590" N, 109° 20' 9.590" E	Harbor
Hainan, Haikou	2	–	HN,HK	19° 58' 50.957" N, 110° 14' 20.835" E	Urban
Hainan, Danzhou	4	–	HN,DZ	19° 31' 52.701" N, 109° 22' 26.660" E	Urban
Hainan, Wenchang	2	–	HN,WC	19° 42' 43.054" N, 110° 45' 9.587" E	Urban
Hainan, Dongfang	10	–	HN,DF	18° 57' 7.445" N, 108° 50' 32.886" E	Urban
Hainan, Sansha	16	6	HN,SS	16° 50' 15.699" N, 112° 20' 14.769" E	Urban

### Microsatellite sequence screening and mtDNA amplification

According to the principles of high conservation and polymorphism, 9 and 10 microsatellite loci for *R*. *tanezumi and R. norvegicus* were screened. Sequences and primers were obtained from Genebank and the literature (Supplementary Table S1), and were synthesized by Biotech Bioengineering (Shanghai) Co.,shanghai, China. The reaction system included 50 μL and contained 10 µL PCR buffer, 10 ng DNA, 0.25 U Taq enzyme (10 p.m./µL, TaKaRa), 10 μM dNTPs (2.5 mM, TaKaRa) and approximately 20 μM of each primer. The 5′ends of the forward primers were labeled with fluorescent dyes (FAM, HEX and ROX). The cycling conditions were as follows: 5 min at 94°C; 10 cycles of 95°C for 30 s, 60°C for 40 s, and 72°C for 50 s; 27 cycles of 94°C for 30 s, 53°C for 40 s, and 72°C for 50 s, 72°C for 10 min. Formamide was then mixed with the internal lane standards at a volume ratio of 100:1, 15 uL was taken and mixed with 10-fold diluted PCR products. Capillary electrophoresis was then performed using a 3730XL sequencer, and the raw data obtained from the sequencer were analyzed using Fragment (Plant) analysis software in Genemarker to obtain fragment sizes.

Mitochondrial sequences of *R. tanezumi* and *R. norvegicus* were amplified using coxl, D-LOOP and ND4 primersand. the detailed primer information is provided in Supplementary Table S1. The amplification system was configured in a thermal cycler with a total volume of 50 μL using genomic DNA as template: 5 μL of template DNA, 2.5 μL of each primer (10 μM) and 40 μL of Ex Taq (TaKaRa Taq). The PCR amplification program was set as follows: predenaturation at 95°C for 3 min, followed by 35 cycles of denaturation at 95°C for 30 s, annealing at 55°C (coxl/ND4/D-LOOP) for 30 s and elongation at 72°C for 1 min, with a final extension at 72°C for 10 min. The PCR reaction products were separated by 1.2% agarose gel electrophoresis, and then imaged by UV gel imager (ClinX, GenoSens 2150,shanghai, China). Samples exhibiting clear, singular positive bands were forwarded to the Institute of Bioengineering in Shanghai, China, for bidirectional sequencing. Blast (https://blast.ncbi.nlm.nih.gov/Blast.cgi) was used to confirm whether they were the target sequences.

### Genetic diversity analysis based on microsatellite and mtDNA

PIC Calc 0.6 was used to calculate polymorphic information content (PIC) values per microsatellite locus for each sample of *R. tanezumi* and *R. norvegicus* based on the microsatellite data (Nagy et al., 2012). Popgene 1.32 (Rousset, 2008) was used to calculate diversity indices including number of different alleles (Na), number of effective alleles (Ne), expected genetic diversity (He), observed heterozygosity (Ho), Shannon Wiener Diversity Index I) and inbreeding coefficient (F_IS_) for each microsatellite locus in both species rat. Based on the mitochondrial data, nucleotide diversity indices that included number of samples S), number of haplotypes H), haplotype diversity index (Hd), and nucleotide diversity (Pi) were calculated for all populations using DNAsp 6.0 software (Rozas et al., 2017).

### Population structure analysis based on microsatellite and mtDNA

Fisher’s exact test with Bonferroni correction based on microsatellite data was used to determine possible deviations from Hardy-Weinberg equilibrium (HWE) and heterozygosity defects (Cristescu et al., 2009). Exact *p*-values were estimated using the Markov chain algorithm with 10,000 dememorizations, 500 batches, and 5,000 iterations per batch (Slatkin, 1995). Pairwise differences between populations and AMOVA analyses based on allele frequencies were analysed using Arlequin software 3.5.1.3 (Slatkin, 1995; Excoffier and Lischer, 2010). Genetic variation was evaluated through Analysis of Molecular Variance (AMOVA) at three distinct levels, aiming to capture genetic diversity and structure among various positional groups within the same rat species. Additionally, the fixation index (F_ST_) for each population was computed, relying on the mean of pairwise differences between populations. (Goudet, 2001). The Unweighted Pair Group Method with Arithmetic Mean (UPGMA) phenogram was built using NTsys software 2.10e (Piry et al., 2017). Alternatively, the discriminant analysis of principal components (DAPC) analyses were performed by the R package “adegenet 1.3" (Jombart, 2008).

### Migration analysis and haplotype diversity

Simultaneously, potential migration routes were reconstructed utilizing the R package “divMigrate,” configuring the number of bootstrap replicates to 3, setting the alpha value to 0.05, opting for the Nm method for the migration statistic, and establishing the filter threshold value at 0.25. (Kharzinova et al., 2016). Statistical assessments for neutral detection, encompassing Fu’s Fs and Tajima’s D, were executed utilizing mitochondrial data with Arlequin 3.5.2.2. The analysis involved plotting observed pairwise nucleotide differences between mtDNA haplotypes against their corresponding expected frequencies. Furthermore, the construction of a TCS network, elucidating haplotype relationships among rodent populations, was accomplished using popART software and was grounded in mitochondrial data. (Leigh and Bryant, 2015).

## Results

### Genetic diversity analysis based on microsatellite and mtDNA

A total of 14 rat populations were sampled from urban, harbor, and rural areas of southeastern coast of China (Table 1; Figure 1). Each of the breeding site buffer zones was isolated from each other, indicating the independence of each population. In addition, 16 pairs of microsatellite markers were obtained from previous studies, all of which were highly polymorphic, and were therefore selected for microsatellite genotyping (Supplementary Table S1). The PIC values for each locus ranged from 0.707 to 0.939, and nearly all selected markers were highly informative (PIC values > 0.5) according to (Ditta et al., 2018) definition of PIC values. Microsatellite results showed average number of alleles (Na) per R. tanezumi population ranged from 3.000 ± 1.323 to 14.200 ± 3.645, and the allele numbers of Jiangsu and Guangdong regions were significantly higher (Table 2). Shannon index of the two provinces was the highest. The observed heterozygosity (Ho) values of all areas ranged from 0.685 ± 0.243 to 0.343 ± 0.340, and were lower than the expected heterozygosity (He) values (ranging from 0.869 ± 0.044 to 0.500 ± 0.258). The FIS values for each population ranged from −0.026 to 0.218, and rat populations in both the Jiangsu and Guangdong regions showed significant deviations from HWE, indicating that these populations contain varying degrees of inbreeding and heterozygote deficiency. The observed Na value interval in the *R. norvegicus* population ranged from 2.917 ± 0.996 to 11.333 ± 2.570, with the largest Na values in the Hainan and Guangdong populations and the smallest Na values in the Zhejiang population. The mean Ho value (0.552 ± 0.179) was significantly lower than the mean He value (0.827 ± 0.067) in the five regional rat populations except Zhejiang, and the Ho value (0.599 ± 0.387) was greater than the He value (0.555 ± 0.218) in the Zhejiang population. The FIS values for each population ranged from −0.026 to 0.218, and the FIS values of Hainan, Guangdong and Guangxi populations were significantly different from HWE values.

**TABLE 2:** Genetic variation of microsatellite loci between *Rattus norvegicus* and *Rattus tanezumi* from coastal cities in the southeastern coast of China.

Species	Location	Na	Ne	I	Ho	He	HEW	F_IS_
*Rattus tanezumi*	Jiangsu	14.200 ± 3.645	7.000 ± 2.734	2.162 ± 0.405	0.580 ± 0.171	0.829 ± 0.088	0.410^***^	0.305^***^
	Guangdong	9.100 ± 2.234	5.273 ± 1.723	1.858 ± 0.277	0.429 ± 0.167	0.792 ± 0.064	0.622^***^	0.400^***^
	Guangxi	5.700 ± 1.160	3.319 ± 0.832	1.397 ± 0.210	0.685 ± 0.243	0.682 ± 0.077	0.013^***^	−0.081
	Hainan	4.889 ± 2.261	3.436 ± 1.841	1.235 ± 0.642	0.530 ± 0.334	0.594 ± 0.280	0.326	0.249^**^
	Fujian	3.000 ± 1.323	2.479 ± 1.115	0.886 ± 0.510	0.343 ± 0.340	0.500 ± 0.258	0.498	0.186
	Zhejiang	3.100 ± 0.876	3.000 ± 0.903	1.074 ± 0.304	0.600 ± 0.460	0.638 ± 0.110	0.267	0.400
*Rattus norvegicus*	Jiangsu	9.416 ± 2.234	6.350 ± 1.648	1.987 ± 0.277	0.683 ± 0.161	0.859 ± 0.057	0.188	0.213***
	Guangdong	11.250 ± 3.223	6.447 ± 1.659	2.054 ± 0.286	0.589 ± 0.129	0.851 ± 0.048	0.386**	0.296***
	Guangxi	10.083 ± 2.021	6.755 ± 1.868	2.056 ± 0.241	0.521 ± 0.146	0.869 ± 0.044	0.624***	0.280***
	Hainan	11.333 ± 2.570	6.350 ± 1.645	2.066 ± 0.278	0.478 ± 0.211	0.854 ± 0.054	0.573**	0.420***
	Fujian	6.333 ± 1.303	3.516 ± 1.216	1.425 ± 0.313	0.489 ± 0.248	0.703 ± 0.131	0.33	0.358***
	Zhejiang	2.917 ± 0.996	2.469 ± 0.809	0.913 ± 0.402	0.599 ± 0.387	0.555 ± 0.218	−0.109	−0.117

***p < 0.001; **p < 0.01; *p < 0.05.

Na, number of different alleles; Ne, number of effective alleles; He, expected genetic diversity expected; Ho, observed heterozygosity; I, shannon index; HEW, Hardy-Weinberg equilibrium; Fis, inbreeding coefficient.

### Population structure analysis based on microsatellite and mtDNA

**FIGURE 2:**
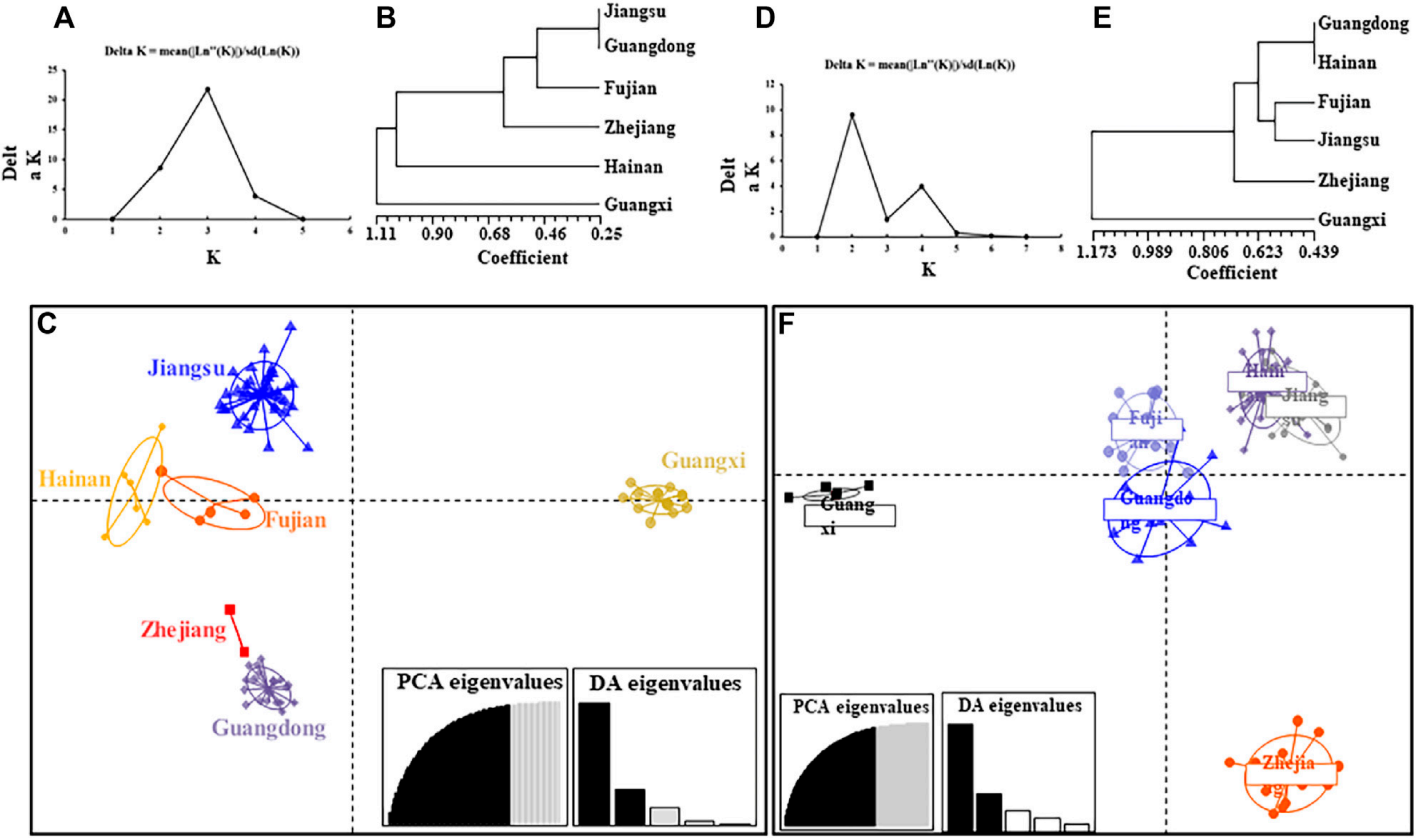
The population structure of *Rattus norvegicus* and *Rattus tanezumi* from six regions in southeastern coast of China based on microsatellite loci. **(A)** Best molecular evolutionary model identified by Bayesian information criteria; **(B)** UPGMA cluster tree analysis of all *R. norvegicus* populations; **(C)** principal components (DAPC) analysis of all *R. norvegicus* populations, and 86.4% of variation was explained by 50 PCs; **(D)** Best molecular evolutionary model identified by Bayesian information criteria; **(E)** UPGMA cluster tree analysis of all *Rattus tanezumi* populations; **(F)** DAPC analysis of all *Rattus tanezumi* populations, and 86.4% of variation was explained by 50 PCs.

Given the significant deviation from Hardy-Weinberg equilibrium (HWE) and the heterozygote defect, it was decidedthat there was further structural refinement among all samples. This study stratified all populations of *R. tanezumi* and *R. norvegicus* into three and two genetically distinct clades, respectively, employing UPGMA cluster tree analysis. (Figures 2B, E). In the cluster analysis of *R. tanezumi* populations, Guangxi and Hainan each constituted distinct branches, while Jiangsu, Guangdong, Fujian, and Zhejiang clustered together, forming another branch. In the Discriminant Analysis of Principal Components (DAPC), all populations exhibited proximity, except for the Guangxi population. Conversely, *R. norvegicus* populations displayed distinct genetic structures, with Guangdong, Hainan, Fujian, Jiangsu, and Zhejiang forming one cohesive group, while Guangxi stood as an isolated group.

The AMOVA results (Table 3) showed that among the different levels of genetic variation in the *R. tanezumi* population, 23.40% of the variation came from differences among individuals within the population; 23.86% of the variation in the *R. norvegicus* population came from differences among individuals within the population. Paired F_ST_ analysis among various regions revealed significant (*p* < 0.05) genetic differentiation of *R. tanezumi* samples from Fujian and Zhejiang compared to populations from other regions, with values ranging from 0.006 to 0.154. *Rattus norvegicus* populations showed moderate to high genetic differentiation in all regional populations (ranging from 0.040 to 0.306) (Table 4).

**TABLE 3:** Analysis of molecular variance (AMOVA) results of *Rattus norvegicus* and *Rattus tanezumi* from the southeastern coast of China.

Species	Source of variation	d.f	Sum of squares	Variance componets	Percentage of variation	Fixation index
*Rattus tanezumi*	Among groups	2	24.192	0.246 Va	11.20	F_CT_ = 0.112
	Among populations	3	11.098	0.052 Vb	2.35	F_SC_ = 0.026^*^
	Within groups					
	Among individuals	82	198.090	0.515 Vc	23.40	F_IS_ = 0.271^***^
	Within populations					
	Total	175	355.381	2.199		
*Rattus norvegicus*	Among groups	2	88.403	0.301 Va	6.9	F_CT_ = 0.069
	Among populations	3	30.472	0.268 Vb	6.14	F_SC_ = 0.066^***^
	Within groups					
	Among individuals	105	507.544	1.041 Vc	23.86	F_IS_ = 0.274^***^
	Within populations					
	Total	221	931.919	4.362		

**TABLE 4:** Differentiation coefficient (FST) analysis results of *Rattus norvegicus* and *Rattus tanezumi* populations from different regions in southeastern coast of China based on mtDNA pairing.

Species		Jiangsu	Guangdong	Fujian	Zhejiang	Guangxi	Hainan
*Rattus tanezumi*	Jiangsu	0.000					
	Guangdong	0.045	0.000				
	Fujian	0.007***	0.006***	0.000			
	Zhejiang	0.041***	0.026***	0.154***	0.000		
	Guangxi	0.148	0.171	0.201	0.074***	0.000	
	Hainan	0.083	0.131	0.175	0.164***	0.204	0.000
*Rattus norvegicus*	Jiangsu	0.000					
	Guangdong	0.306***	0.000				
	Fujian	0.246***	0.141***	0.000			
	Zhejiang	0.235***	0.111***	0.080***	0.000***		
	Guangxi	0.213***	0.102***	0.059***	0.040***	0.000	
	Hainan	0.248***	0.105***	0.050***	0.050***	0.048***	0.000

***p < 0.001; **p < 0.01; *p < 0.05.

### Migration analysis and haplotype diversity

**FIGURE 3:**
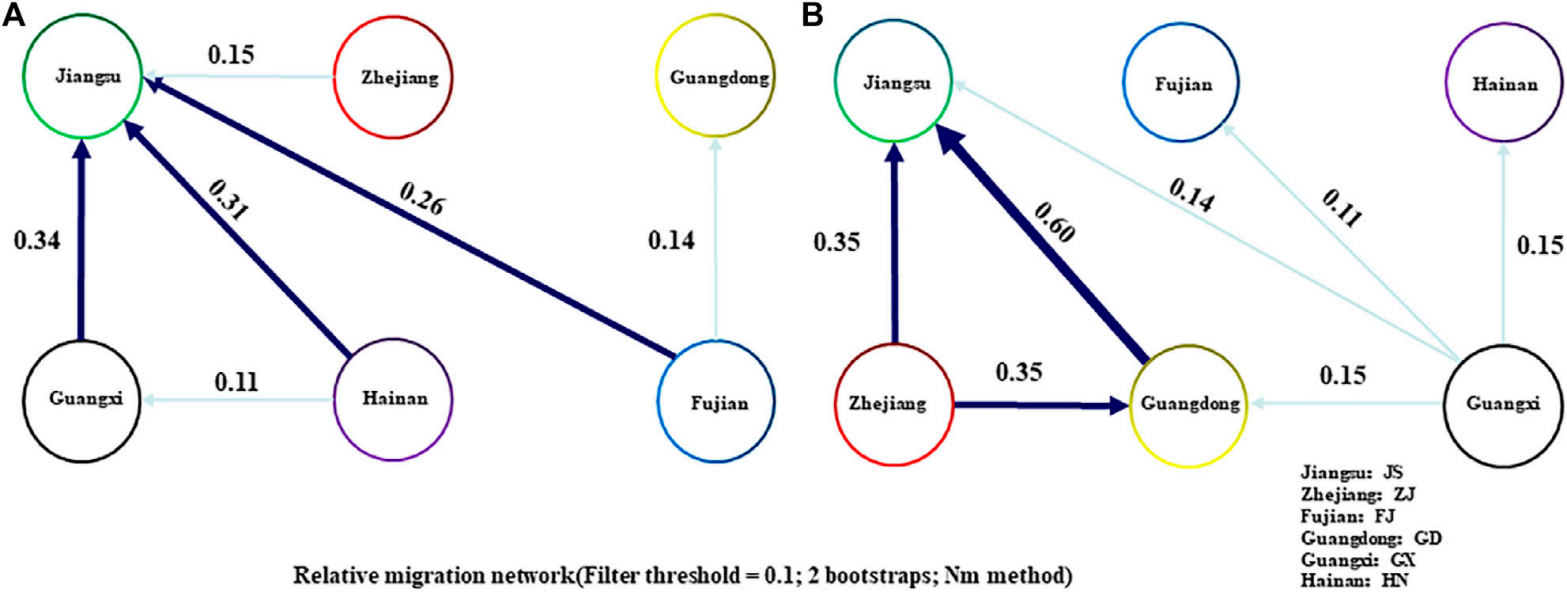
Migration patterns of *Rattus tanezumi* and *Rattus norvegicus* from southeastern coast of. Migration routes are marked with different types of arrows (dark blue: gene flow >0.3; light blue: gene flow <0.3). Diffusion diagrams of the *R. tanezumi* **(A)** and *Rattus norvegicus* **(B)**.

Three mitochondrial sequences of coxl (750bp), D-LOOP (950bp) and ND4 (450bp) were obtained from 188 samples. The polymorphism of the control region of D-LOOP was significantly higher than that of coxl and ND4, but the rapid evolutionary rate may lead to unstable results and a large number of mutations. The coxl sequence with stable evolutionary rate was selected for analysis, and a total of 16 haplotypes were found in each species of *R. tanezumi* and *R. norvegicus*. The highest genetic diversity of the *R. tanezumi* was found in the Jiangsu populations, and the lowest genetic diversity was found in the Fujian populations (Table 5). The nucleotide diversity of *R. norvegicus* population in Fujian was the highest (Pi = 0.01214), while that of *R. norvegicus* population from Guangxi was the lowest (Pi = 0.00200) (Table 5). In the neutral test of Tajima’s D and Fu’s F, *R. norvegicus* was positive in all regions, while *R. tanezumi* was significantly negative (*p* < 0.05) except Guangxi and Zhejiang. Migration patterns were assessed using a divMigrate network representing all rat populations. For gene flow analyses, although both *R. norvegicus* and *R. tanezumi* had migration routes from other regions to the Jiangsu region, their gene flow mobility levels were at low values (Figure 3).

**TABLE 5:** Mitochondrial polymorphism indicators and neutral detection cytochrome c oxidase subunit I (coxl) *Rattus norvegicus* and *Rattus tanezumi* populations from different regions in southeastern coast of China.

	Location	S	H	Hd	Pi	Tajima’s *D*	Fu’s F	Haplotype
*R. tanezumi*	Guangxi	15	5	0.714	0.00647	0.956	0.763	H1(7); H2(1); H3(3); H4(1);H5(4)
	Zhejiang	2	2	-	-	-	-	H06(1), H07(1)
	Fujian	7	1	0.762	0.00132	0.687	1.145	H13(7)
	Guangdong	20	2	0.378	0.00225	−1.873*	−2.366*	H7(19); H8(1)
	Hainan	6	3	0.600	0.00292	−1.295	−1.396	H7(4); H9(1); H10(1)
	Jiangsu	36	6	0.627	0.00772	−1.497*	−2.139*	H7(20); H11(2); H12(8); H14(4); H15(1); H16(1)
*R. norvegicus*	Guangxi	15	2	0.952	0.00200	1.631	1.377	H01(10); H02(5)
	Zhejiang	15	5	2.419	0.00387	0.451	0.414	H01(9); H03(1); H04(3); H05(1); H06(1)
	Fujian	19	4	2.503	0.01214	−2.231**	−3.580**	H01(3); H04(14); H07(1); H08(1)
	Jiangsu	17	7	3.750	0.00720	0.038	−0.734	H01(4); H04(1); H07(1); H09(7); H10(1); H11(2); H12(1)
	Hainan	33	7	2.625	0.00447	0.203	−0.682	H01(21); H03(2); H04(6); H13(1); H14(1); H15(1); H16(1)
	Guangdong	15	3	2.686	0.00443	1.688	1.629	H01(5); H03(8); H04(2)

S, number of samples; H, number of haplotypes; Hd, Haplotype diversity; Pi, Nucleotide diversity.

**FIGURE 4:**
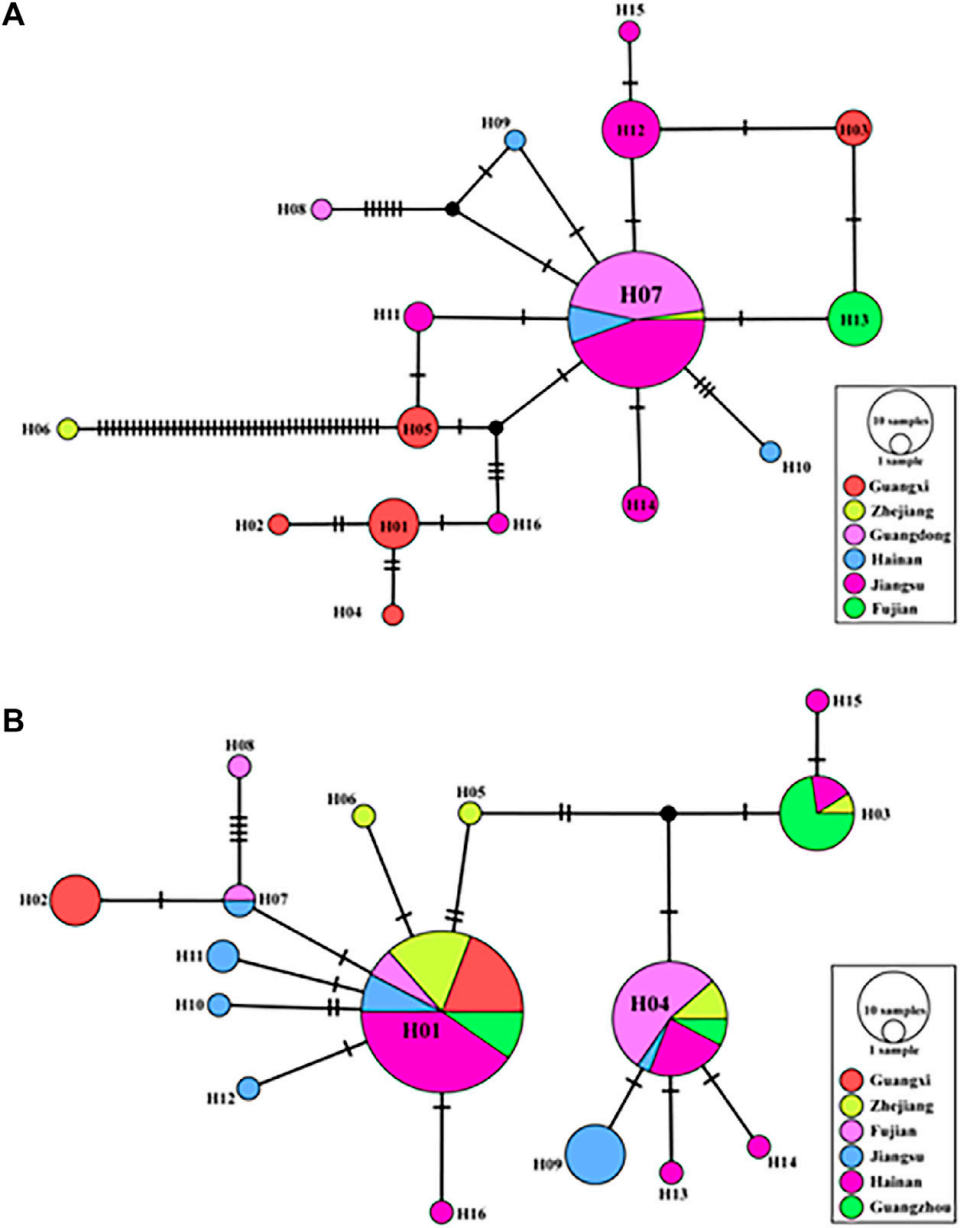
A topography of haplotype Haplotype network based on mitochondrial cytochrome c oxidase subunit I (coxl) sequences in Rattus norvegicus and R. tanezumi from southeastern coast of China. Haplotype network diagrams of the *Rattus tanezumi* **(A)** *R. norvegicus* **(B)**. The solid pie chart represents the observed haplotypes, the circle size represents the number of individuals possessing the haplotype, and the colors represent the different sampling locations. Each black connecting line represents a mutant base step between different haplotypes.

A TCS network was reconstructed using all cox1 sequences (Figure 4). Within the *R. tanezumi* haplotype networks, haplotype 7 (H7), the most prevalent haplotype, predominantly occurred in Jiangsu, Hainan, and Guangdong, shaping a star-like network. The number of haplotypes exhibited a decline in southern Jiangsu and eastern Guangdong, with haplotype 13 (H13) exclusively present in Fujian. The TCS network map of *R. norvegicus* portrayed a star-shaped network structure, predominantly centered on haplotype 1 (H1) and haplotype 4 (H4), prevalent in Guangxi, Zhejiang, Fujian, and Jiangsu. Meanwhile, haplotype 9 (H9), haplotype 10 (H10), haplotype 11 (H11), and haplotype 12 (H12) were confined to distribution solely in Jiangsu.

## Discussion

### Population genetics of *Rattus norvegicus* and *Rattus tanezumi*

The southeastern coast features a warm and humid subtropical marine monsoon climate, transitioning into a tropical monsoon climate. Characterized by mild weather and consistently high annual temperatures in most regions, this climatic profile historically designated it as the primary distribution area for *R. tanezumi* and *R. norvegicus*. Its climatic condition is a suitable breeding ground for rodents, and the main channel of economic and cultural exchange between China and continental Europe is seaport shipping. Therefore, seaports are considered introduction epicentres for invasive alien species (Drake and Lodge, 2004; Early et al., 2016). The combination of adequate shelter, food and water supply has led to a high population of rat in seaports (Himsworth et al., 2013; Johnson and Munshi-South, 2017). The economic development brought by the well-developed trade in coastal cities accelerated local urbanization, making it an important area for the export and migration of rats to other areas.

Of our mitochondrial data showed that *R. tanezumi* populations from Jiangsu and Guangxi possess higher genetic diversity than populationsfrom other regions. Genetic diversity has a significant impact on the evolutionary process can play an important catalytic and driving role at all levels, including population, community and ecosystem levels (Hughes et al., 2008). The same results were obtained from allele (Na) and heterozygosity (He and Ho) analyses in microsatellite analysis. It is therefore hypothesized that there may be an invasion of exotic populations of *R. tanezumi* in Hainan, Zhejiang and Fujian, which may also account for its lack of significant deviation from HEW. This conclusion was also supported by the neutral detection of Tajima’s D and Fu’s F values, which were negative and significant, which may be caused by the recent expansion of the population in *R. tanezumi*.

The findings pertaining to *R. norvegicus* revealed elevated haplotype diversity and nucleotide diversity within the Jiangsu and Hainan populations. This observation suggests the possibility of secondary evolution or interpopulation contact influencing the genetic dynamics in this particular group. (Wenzel et al., 2015). The values of He across different populations varied from 0.555 to 0.869, unequivocally signifying the presence of high polymorphism. Additionally, the Ho values for all populations were consistently lower than their corresponding He values. Only the Zhejiang population had a higher Ho value, indicating that there was no heterozygous deficiency in this population. This may be means that diversity is high in that site. Conversely, the departure from Hardy-Weinberg equilibrium (HWE) observed in both *R. tanezumi* and *R. norvegicus* populations in Guangxi and Guangdong may be attributed to the hot and humid climate, as well as the complex geological environment in southern China. Additionally, the impact of population migration and expansion driven by social development emerges as a crucial influencing factor.

### Genetic structure and dispersal of *Rattus tanezumi* and *Rattus norvegicus*

The results of the three methods of analysis based on microsatellites data showed slightly different results. All samples of the *R. tanezumi* population were divided into three clusters in UPGMA. Hainan and Guangxi were divided into one branch each, and Jiangsu, Guangdong, Fujian and Zhejiang were unified into one branch. The DPCA analysis showed the same results as the cluster analysis, and interestingly, the Jiangsu and Guangdong populations clustered together in the genetic structure of both species of house rat (Figures 2C, F). This suggests that there are some factors that led to the exchange and spread of species between the two areas. The close affiliation between Jiangsu and Guangdong populations was further evident in the haplotype analysis of their mitochondrial sequences. In the cox1 haplotype network of *R. tanezumi*’s mtDNA sequences, the network structure centered around the principal haplotypes in Jiangsu and Guangdong, radiating in a star-shaped pattern. This pattern indicates population expansion in these two regions, aligning with the results of neutral detection. The widespread distribution of H7 haplotypes across populations in all provinces along the southeastern coast suggests that, despite the geographical distance between Jiangsu and Guangdong, they are positioned at the extremities of the southeastern coast, fostering genetic exchange or shared ancestry. Additionally, haplotype analysis of mitochondrial sequences in *R. norvegicus* identified two predominant haplotypes (H1 and H4), with H4 being prominent in Fujian and H1 in Hainan regions Fujian exhibited an expansion in the outcomes of its neutrality test, accompanied by a noteworthy richness in haplotype diversity. The haplotype network diagram further illustrates a connection among rat populations in Fujian, Hainan, and Jiangsu. The coastal regions, particularly in Fujian, are distinguished by mountainous terrains and seascapes, featuring high forest cover and favorable temperatures, creating ideal environments for rat breeding and dissemination. Jiangsu Province, situated in the Yangtze River Economic Zone and east of the Yellow Sea, is intersected by the Yangtze River and Huai River, encompassing two major water systems. With a geographical expanse from north to south, the province displays a hierarchically diverse climate and vegetation. The intricate river trade and railroad transportation network further contribute to the complexity, potentially facilitating the spread of rodents.

Analyzing the history of segregation and gene flow during species differentiation provides valuable insights into the process of their formation. Notably, a substantial level of gene exchange is evident among the populations of *R. norvegicus* in Guangdong, Zhejiang, and Jiangsu, as depicted in the gene flow dispersal map. The discernible trend indicates a movement from Guangdong and Zhejiang towards the Jiangsu region, aligning with the overarching northern expansion pattern observed in *R. norvegicus*. In contrast, the Fujian population is notably geographically isolated from several other regions. Despite this isolation, its high genetic diversity suggests the absence of a significant founder effect, hinting at potential transmission exchanges with Southeast Asia. While *R. norvegicus* was initially considered to have originated from northern China and Mongolia, the intricate genetic dynamics observed in the Fujian population present a more nuanced perspective (Lin et al., 2012; Song et al., 2014). Subsequent analysis of dated fossils discovered in Guangxi Province, China, believed to represent the ancestors of *R. norvegicus*, proposed an origin for the species in present-day southwestern China, around 1.2–1.6 million years ago (Jin et al., 2008). This finding provides an explanation for the observed high genetic diversity of *R. norvegicus* in Fujian and Hainan provinces in southern China. Previous studies have indicated that a significant portion of the genetic structure in *R. norvegicus* is attributed to geographic and environmental heterogeneity (Puckett et al., 2016; Zhao et al., 2020).

*Rattus tanezumi* populations in the Yangtze River basin diverged from rats in central regions such as Yunnan, and Aplin et al. (2011). Reported an expansion of *R. tanezumi* through human shipping 4,000 years ago (Aplin et al., 2011). Guo et al. (2019) reported that *R. tanezumi* may have originated from Yunnan and neighboring Southeast Asia, and relied primarily on maritime shipping for early dispersal (Guo et al., 2019). This lends support to the notion that the ancestors of *R. tanezumi* were transported along the road to the Yangtze River basin during that period, expanding towards the warmer inland and southeastern coastal regions. Simultaneously, population migration also occurred in neighboring provinces such as Guangxi and Guangdong, influenced by anthropogenic factors. It can therefore be speculated that the Guangdong and Jiangsu populations may have originated from the same ancestor. On the flip side, the populations of *R. tanezumi* in Fujian, Hainan, and Zhejiang regions appear to have propagated from both Guangdong and Jiangsu through trade routes. The reduced population genetic diversity in these regions may be attributed to the accumulation of deleterious alleles or mutations linked to invasion and post-colonial expansion (Sakai et al., 2001). Nevertheless, invasive populations, even with reduced genetic diversity, can still successfully colonize and expand in new environments (Tsutsui et al., 2000; Carlson et al., 2014).

### Dispersal ability and dissemination risk of *Rattus tanezumi* and *Rattus norvegicus*

Within China, *R. norvegicus* and *R. tanezumi* emerge as key commensal rodent species. This study reveals a significant resemblance in their distribution patterns with respect to overall genetic structure. Jiangsu province, serving as a crucial pathway within the Yangtze River basin, displays remarkably high genetic diversity. Nevertheless, the observed contrast in genetic diversity between the compared populations of *R. norvegicus* and *R. tanezumi* in Fujian suggests potential distinctions in their dispersal capabilities and pathways. *R. norvegicus* populations exhibit lower dispersal ability over long distances when competing with *R. tanezumi* in the United States (Lack et al., 2013). Over the past half century, *R. norvegicus* has invaded the Xinjiang region with the aid of rail transport and other modern forms of transportation (Liao et al., 2014). Compared to the northwestern expansion of *R. norvegicus*, *R. tanezumi* has expanded northward at a lower rate in recent years. This suggests that *R. tanezumi* is less suitable than the *R. norvegicus* to use modern transportation systems for long-distance dispersal. This may be due to the different daily routines of these two parasitic rodents around humans. Although they both inhabit human housing and various types of buildings, the primary range of *R. tanezumi* is in high-rise spaces, as opposed to the *R. norvegicus*, which results in easier migration and dispersal via transportation.

Worth noting is the impact of the migration of commensal rodents, including the spread of rodent-borne diseases carried by these two species of rodents that accompany the migration of populations. Urban environments have proven to be favorable for rat population growth and associated transmission of zoonoses (Tian et al., 2018; Clement et al., 2019). *Rattus tanezumi* and *R. norvegicus* are natural hosts of hantaviruses, and they are far more mobile than other hantavirus hosts. This has led in many cases to correlations between host taxa and their associated hantaviruses (Plyusnin and Sironen, 2014; Zuo et al., 2018). Through phylogenetic analysis of the high concordance between hantavirus and its host (*R. norvegicus*), Lin et al. (2012) found that the global spread of Orthohantavirus was a consequence of the outward expansion of *R. norvegicus* in China (Lin et al., 2012). Domestic reports have also described a high similarity of 98.0%–99.0% between the Hantaan strain from Ningde port and the strain from Shandong (Lin et al., 2012). It is interesting to note that the increase in mild HFRS coincides with the rapid socioeconomic development that began in 1978 (Lin et al., 2012). In addition to the risk of hantavirus transmission, plague deserves attention as an important rodent-borne disease, with bubonic pest epidemics occurring from western Yunnan to the eastern part of the country in the 26 years from 1986 to 2005 (Li et al., 2008). This is similar to our findings that the two symbiotic rats expanded from coastal areas to central China through shipping and modern transportation, respectively. Collectively this shows that the virus creates a broader risk of transmission during its activity with the host, and according to previous studies the hosts of hantavirus are mostly *R. norvegicus*, but the virus sequences were found to be highly similar in *R. tanezumi* and *R. norvegicus*, so there is also the possibility of cross-species transmission in bothrat species (He et al., 2021). Currently, fast transportation has started to provide these house rat more opportunities for long-distance transmission and various rodent-borne diseases have been transmitted by rodents over relatively long distances. Previously this expansion of the geographic range of commensal rat species was considered to be the result of human activities, therefore detailed biological and anthropological investigations are necessary to provide traceable information for future infectious diseases.

## Conclusion

The results of this study further support the dispersal trend of *R. tanezumi* populations in the southeastern coastal areas of China from the southern regions or coastal routes to the inland Yangtze River basin. Historically, the population spread towards the warmer interior and the south, and gradually to the southeast. Subsequently, with the development of global warming and rail transport, the overall spread was toward the north. *Rattus norvegicus* has a similar trend of invasion from south to north, but its rate of spreadhigher. Many specific dispersal mechanisms still need to be further studied. This study provides a more refined population structure of *R. tanezumi* and *R. norvegicus* populations in the southeastern coastal region, which can provide new perspectives and assist in the prevention and control of zoonotic disease transmission to assist in the development of more effective rodent control programs.

## Data Availability

The datasets presented in this article are not readily available because of policy and privacy restrictions. Requests to access the datasets should be directed to the corresponding authors.

## Abbreviations

*R. norvegicus*, *Rattus norvegicus*; *R. tanezumi*, *Rattus tanezumi*; HFRS, hemorrhagic fever with renal syndrome; PIC, PIC polymorphism information content; Na, Number of allele; Ne, effective number of allele; Ho, observed heterozygosity; He, expected heterozygosity; I, shannon index; FIS, inbreeding coefficient; F_ST_, genetic differences among populations; HWE, hardy-weinberg equilibrium; AMOVA, analysis of molecular variance; H, haplotype number; Hd, haplotype diversity index; Nm, number of migrants; DAPC, discriminant analysis of principal components; π, nucleotide diversity; mtDNA, mitochondrial DNA; PCR, polymerase chain reaction.
